# The multi-kinase inhibitor afatinib serves as a novel candidate for the treatment of human uveal melanoma

**DOI:** 10.1007/s13402-022-00686-5

**Published:** 2022-07-04

**Authors:** Wenying Shu, Xue Zhu, Ke Wang, Svetlana Cherepanoff, R. Max Conway, Michele C. Madigan, Hong Zhu, Ling Zhu, Michael Murray, Fanfan Zhou

**Affiliations:** 1 Department of Pharmacy, Affiliated Cancer Hospital & Institute of Guangzhou Medical University, Guangzhou, 511400 Guangdong Province China; 2 Key Laboratory of Nuclear Medicine, Ministry of Health, Jiangsu Key Laboratory of Molecular Nuclear Medicine, Jiangsu Institute of Nuclear Medicine, Wuxi, 214063 Jiangsu Province China; 3 SydPath, Department of Anatomical Pathology, St Vincent’s Hospital, Darlinghurst, NSW 2010 Australia; 4 Ocular Oncology Unit, Sydney Eye Hospital and The Kinghorn Cancer Centre, Sydney, NSW 2006 Australia; 5 Save Sight Institute, The University of Sydney, Sydney, NSW 2006 Australia; 6 School of Optometry and Vision Sciences, University of New South Wales, Sydney, NSW 2006 Australia; 7 Zhejiang Province Key Laboratory of Anti-Cancer Drug Research, College of Pharmaceutical Sciences, Zhejiang University, Hangzhou, 310058 Zhejiang Province China; 8 Sydney Pharmacy School, Faculty of Medicine and Health, The University of Sydney, Camperdown, NSW 2006 Australia

**Keywords:** Uveal melanoma, HER2, Afatinib, Cell cycle arrest, Anti-metastatic activity

## Abstract

**Purpose:**

Uveal melanoma (UM) is the most common intraocular malignancy in adults with a poor prognosis and a high recurrence rate. Currently there is no effective treatment for UM. Multi-kinase inhibitors targeting dysregulated pro-tumorigenic signalling pathways have revolutionised anti-cancer treatment but, as yet, their efficacy in UM has not been established. Here, we identified the multi-kinase inhibitor afatinib as a highly effective agent that exerts anti-UM effects in in vitro*,* ex vivo and in vivo models.

**Methods:**

We assessed the anti-cancer effects of afatinib using cell viability, cell death and cell cycle assays in in vitro and ex vivo UM models. The signaling pathways involved in the anti-UM effects of afatinib were evaluated by Western blotting. The in vivo activity of afatinib was evaluated in UM xenograft models using tumour mass measurement, PET scan, immunohistochemical staining and TUNEL assays.

**Results:**

We found that afatinib reduced cell viability and activated apoptosis and cell cycle arrest in multiple established UM cell lines and in patient tumour-derived primary cell lines. Afatinib impaired cell migration and enhanced reproductive death in these UM cell models. Afatinib-induced cell death was accompanied by activation of STAT1 expression and downregulation of Bcl-xL and cyclin D1 expression, which control cell survival and cell cycle progression. Afatinib attenuated HER2-AKT/ERK/PI3K signalling in UM cell lines. Consistent with these observations, we found that afatinib suppressed tumour growth in UM xenografted mice.

**Conclusion:**

Our data indicate that afatinib activates UM cell death and targets the HER2-mediated cascade, which modulates STAT1-Bcl-xL/cyclin D1 signalling. Thus, targeting HER2 with agents like afatinib may be a novel therapeutic strategy to treat UM and to prevent metastasis.

**Supplementary Information:**

The online version contains supplementary material available at 10.1007/s13402-022-00686-5.

## Introduction

Uveal melanoma (UM) accounts for ~85% of ocular melanomas and 3% of all melanomas in humans. The National Organization for Rare Diseases estimates that the incidence of UM is ~5–7 per million in the general population [1, 2] Although UM has a relatively low incidence, its mortality rate is high and up to 50% of patients ultimately succumb to metastases [2, 3]. Patient survival has remained poor, presumably due to silent hematogenous systemic micro-metastases that are present prior to the diagnosis of clinically evident ocular symptoms. Once metastases are established and are of detectable size, death occurs within 6–12 months [4–7].

Currently, front-line treatment for UM includes radiotherapy, phototherapy and surgery, but vision impairment, blindness or eye removal are common clinical consequences following these treatments [8]. Because the aetiology, genetic associations and clinical behaviour of UM are distinct from cutaneous melanoma, drugs that are effective in cutaneous melanoma are ineffective in UM patients, especially those with metastatic disease. Indeed, there are no drugs that have been found to be effective in treating primary or metastatic UM, including those that target multiple signalling cascades dysregulated in UM [9–14].

Most UM tumours exhibit mutations in genes encoding the G protein-alpha subunits GNAQ or GNA11 that activate the mitogen-activated protein kinase (MAPK) and phosphoinositide-3 kinase (PI3K)/Akt signalling pathways [15]. Initial studies implicated the Epidermal Growth Factor Receptor (EGFR or ErbB1) in UM tumour proliferation and metastasis [16, 17]. The EGFR has been reported to be expressed in a small proportion of UM cell lines and tumours [17–19], and the ligand EGF has been reported to activate the phosphorylation of EGFR and its downstream mediator AKT in EGFR-expressing cell lines [18]. Furthermore, scleral invasion activity has been associated with higher vitreal EGF concentrations in UM patients [16]. However, some investigators found no association between EGFR expression and UM development [20, 21]. Scholes et al. reported that EGFR immunoreactivity was restricted to macrophages [22]. Moreover, clinical trials of the multi-kinase inhibitor (MKI) gefitinib that targets the EGFR have been unsuccessful in UM [18, 23] and a number of studies has questioned the functional importance of EGFR in UM [24]. Therefore, the clinical relevance of EGFR in UM remains controversial.

There are four isoforms in the ErbB lineage of proteins (ErbB1–4) that function as homo- and heterodimers [25]. The dimerization of EGFR and ErbB2 (HER2) is associated with a poor prognosis and with cell invasion in a range of tumours [26]. HER2 signalling regulates a number of important targets with clinical roles in tumorigenesis. From a phosphor-proteomics analysis of cell lines in which HER2 was overexpressed, tyrosine phosphorylation of 198 proteins, including STAT1, was found to be increased [27]. STAT1 has been shown to regulate cell cycle progression by modulating the expression of cyclin D1 in tumour cells [28, 29].

HER2 is expressed in UM tumour cells [30]. In the present study, we evaluated several MKIs with the capacity to inhibit ErbBs for their effect on the viability of UM cells. The principal finding is that afatinib, which is an established inhibitor of EGFR, HER2 and HER4, serves as an effective agent that exerts anti-UM activity in a range of in vitro*,* ex vivo and in vivo models. Thus, afatinib emerges as a new candidate for clinical evaluation in UM patients.

## Materials and methods

### Reagents

Dulbecco’s Modified Eagle Medium (DMEM), Roswell Park Memorial Institute Medium (RPMI-1640), Fetal Bovine Serum (FBS), Insulin-Transferrin-Selenium (ITS), Penicillin-Streptomycin (P/S) and L-Glutamine were purchased from Thermo Scientific (Lidcombe, NSW, Australia). Giant cell tumour (GCT) conditioned medium was obtained from United Biosciences (Carindale, QLD, Australia). Dimethyl sulfoxide (DMSO), L-glutamic acid monosodium salt hydrate and thiazolyl blue tetrazolium bromide (MTT) were purchased from Sigma-Aldrich (Castle Hill, NSW, Australia). The MKIs afatinib, bosutinib, cediranib, foretinib, lapatinib, erlotinib, gefitinib, neratinib, pelitinib, vandetanib, crizotinib, sorafenib and sunitinib were obtained from Selleck Chemicals (Houston, TX, USA) [31]; all MKIs were dissolved in DMSO. Antibodies directed against cyclin D1 (Cat. #: 55506), Akt (pan, Cat. #: 4685), Phospho-Akt (Ser473, Cat. #: 4060), p44/42 MAPK (Erk, Cat. #: 4695), phosphor-p44/42 MAPK (Thr202/Tyr204, Cat. #: 4370), HER2/ErbB2 (Cat. #: 4290), phospho-HER2/ErbB2 (Tyr1196, Cat. #: 6942), phospho-PI3 Kinase p85 (Tyr458)/p55 (Tyr199) (E3U1H, Cat. #: 17366), PI3 Kinase p85 (19H8, Cat. #: 4257), STAT1 (D1K9Y, Cat. #: 14994), Bcl-xL (54H6, Cat. #: 2764), Bax (D2E11, Cat. #: 5023), phospho-EGF Receptor (Tyr1173) (53A5, Cat. #: 4407), GAPDH (D16H11, Cat. #: 5174) and EGF Receptor (D38B1, Cat. #: 4267) were purchased from Cell Signalling Technology (Danvers, MA, USA). A FITC-Annexin V and Propidium Iodide (PI) apoptosis detection kit was purchased from BD Bioscience (North Ryde, NSW, Australia). PVDF membranes were purchased from Merck Millipore (Bayswater, VIC, Australia). An anti-β-actin antibody was obtained from Sigma-Aldrich. Goat anti-mouse and anti-rabbit IgGs conjugated with horseradish peroxidase (HRP) were obtained from Bio-strategy delivery technology (Tullamarine, VIC, Australia).

### UM cell lines

The human Mel202 UM cell line was kindly provided by Prof. B. Ksander (Schepens Eye Research Institute, Boston, MA, USA). The 92.1 cell line was a gift from Prof. M.J. Jager (Leiden University Medical Center, Leiden, Netherlands) and the OMM-1 cell line was a gift from Prof. G.P. Luyten (Erasmus University, Rotterdam, Netherlands). The C918 cell line was purchased from BioScientific (Gymea, NSW Australia) and the BeNa Culture Collection (Beijing, China). All cell lines were authenticated in-house or by the respective commercial suppliers and routinely checked for mycoplasma contamination every 6 months using a MycoAlert Mycoplasma Detection kit (Lonza, Mount Waverley, VIC Australia). They were always negative. C918, Mel202 and 92.1 cells were cultured in RPMI-1640 medium supplemented with 10% heat-inactivated FBS (v/v), 1% P/S and 1% L-Glutamine (Thermo Scientific, Lidcombe, NSW, Australia). OMM-1 cells were cultured in DMEM medium supplemented with 10% heat-inactivated FBS (v/v), 1% P/S and 1% L-Glutamine. All cell lines were maintained in a humidified incubator (5% CO_2_) at 37 °C and used within 20 passages after thawing.

### Cytotoxicity assay

UM cells were cultured in 96-well plates (2 × 10^4^ cells/well) for 24 h. Subsequently, cells were treated with MKIs (10 μM in 0.1% DMSO) in RPMI-1640 or DMEM containing 1% FBS (v/v) for 24 h; 0.1% DMSO was used as the negative control. Following treatments, cells were incubated with MTT (0.5 mg/ml) in the dark for 3 h and then washed with phosphate-buffered saline (PBS, 0.154 M NaCl, 0.001 M KH_2_PO_4_, 0.003 M Na_2_HPO_4_; pH 7.4). Next, the cells were treated with DMSO and the plate was shaken for 10 min at room temperature. Absorbance values were measured at 550 nm in a microplate reader (Model 680, Bio-Rad, Gladesville, NSW, Australia) [32, 33]. IC_50_ values for MKIs were estimated by non-linear regression of percentage cell survival vs drug concentration data (GraphPad Prism 7.0; San Diego, CA).

### Annexin V/PI flow cytometry assay

UM cells were treated with MKIs (5 μM in 0.1% DMSO) in RPMI-1640 or DMEM containing 1% FBS (v/v) for 24 h at 37 °C; 0.1% DMSO was used as the negative control. Next, the cells were collected and stained with PI and annexin V-FITC for 20 min at room temperature [34, 35] and analysed for apoptosis and necrosis using a Guava easy®cyte flow cytometer (Merck Millipore, Bayswater, VIC, Australia).

### Cell cycle analysis

UM cells were treated with MKIs (5 μM in 0.1% DMSO) in RPMI-1640 or DMEM containing 1% FBS (v/v) for 12 h at 37 °C; 0.1% DMSO was used as the negative control. Next, the cells were then harvested, washed twice with PBS and fixed in ice-cold 70% ethanol (v/v) for 16 h at 4 °*C. *Prior to the analysis, cells were washed with PBS and stained with PI for 30 min in the dark at 37 °C. The samples were analysed using a Guava easy®cyte flow cytometer.

### Cell migration assay

UM cells were cultured on 96-well ImageLock™ microplates (Sartorius Australia, Dandenong, VIC; 5 × 10^4^ cells/well) for 24 h. Next, scratches (‘wounds’) were made by a Wound Maker™ (Sartorius Australia) after which the cells were washed twice with PBS. Next, the cells were treated with MKIs (5 μM in 0.1% DMSO) in RPMI-1640 or DMEM containing 1% FBS (v/v) for 24 h; 0.1% DMSO was used as the negative control. The microplates were placed in an Essen IncuCyte S3^®^ instrument^,^ (Sartorius, Dandenong South, VIC, Australia) and incubated at 37 °C for 24 h. Images were taken at 10x magnification at 2 h intervals. Image J software (National Institutes of Health, USA) with Colony Counter Plugin was used to estimate the leading edge of the cell population. The migration rate was calculated as described previously [36].

$$Migration\ Rate\%=\left[\frac{\mathrm{Area}\left(\mathrm{initial}\right)-\mathrm{Area}\left(\mathrm{final}\right)}{\mathrm{Area}\left(\mathrm{initial}\right)}\right]\times 100\%$$

Area (initial) is the area of the scratch measured immediately after scratching (t = 0 h). Area (final) is the area of the wound measured 24 h after the scratch was applied.

### Colony formation assay

Cells were treated with MKIs (10 μM) or 0.1% DMSO for 24 h and then sub-cultured in 12-well plates (200 cells/well) for 6–8 days. On the day of analysis, the cells were stained with 0.01% crystal violet (w/v) and then assessed for colony growth. A colony is defined as a cluster of at least 50 cells determined microscopically. The plates were photographed in an Essen IncuCyte S3^®^ instrument, using the whole-well scan mode at 4x magnification. Image J software was used to estimate the leading edge of the cell population.

### Western blotting

UM cells were harvested and treated with lysis buffer containing NP-40 (1% IGEPAL, 50 mM Tris and 150 mM NaCl, pH 7.8 containing protease inhibitors). The lysates were centrifuged at 15,000 rpm (10 min, 4 °C). Protein samples were denatured and separated by electrophoresis. After transfer to PVDF membranes, the blots were incubated with 5% non-fat milk (in PBST) for 30 min at room temperature. The blots were incubated with a primary antibody at 4 °C overnight with orbital shaking and then washed three times with PBST. Next, the blots were incubated with a secondary antibody for 1 h at room temperature and then with a chemiluminescent substrate (SuperSignal West Pico, Thermo Scientific, Lidcombe, NSW, Australia). The signals were visualized using ImageQuant LAS500 (GE health care, Silverwater, NSW, Australia).

### Primary UM tumour-derived cell lines

Human UM tumour samples were obtained as approved by St. Vincent’s Hospital Sydney Human Ethics Committee and experiments were performed in strict accordance with the relevant guidelines and regulations. After surgical removal of the UM tumour tissues, the samples were washed three times with PBS (pH 7.4) and processed for cell isolation within 24 h. Trypsin-EDTA was applied to separate the cells that were collected in RPMI-1640 medium containing 20% FBS (v/v), 1% L-glutamine, 1% P/S, 1% ITS and 2% GCT. Primary UM tumour-derived cell lines were maintained at 37 °C with 5% CO_2_ and used between passages 2 to 5 in subsequent experiments. The three patient UM tumour-derived cell lines were characterised by immunostaining using anti-Tyrp1 (a melanocyte- and melanoma-specific marker) and anti-melanoma (a melanoma-specific marker) antibodies (Supplementary Fig. 1).

### UM xenograft mouse model

Animal ethics approval was obtained from the Laboratory Animal Ethics Committee of Jiangsu Institute of Nuclear Medicine (Wuxi, China) and all animal experiments were performed in strict accordance with the relevant guidelines and regulations. C918 cells were mixed with Matrigel in a 2:1 (v:v) ratio and injected subcutaneously into BALB/c nude mice (5 weeks old; male; Chang Zhou Cavens Laboratory Animal Co., Ltd., Changzhou, China). Tumour volumes were measured every three days using callipers and treatments were started when a tumour volume of ~100 mm^3^ was reached. The mice were randomly divided into two groups to receive either afatinib (15 mg/kg; *n* = 12) or vehicle (*n* = 10) by intraperitoneal injection once daily for 16 days. Body weights and tumour volumes were measured every four days. The volumes of tumours were calculated as (a × b^2^)/2, where a and b were the length and width of the tumours, respectively. At the end of the treatment, the mice were anesthetized by intraperitoneal injection of 5 ml/kg 1% pentobarbital sodium salt. The tumours were removed, weighed and photographed. Finally, the tumour samples were fixed in 4% paraformaldehyde for pathological examination.

### Positron emission tomography (PET) scan

To prepare [^68^Ga] Ga-NOTA-PRGD2 tracer, fresh ^68^Ga activity was eluted from a ^68^Ge/^68^Ga generator with 0.05 M HCl; 1.5 ml fractions were collected. The radioactive fraction (~5.26 MBq) was added to 1 M sodium acetate buffer containing 50 μg NOTA-PRGD2. The mixture was then heated at 97 °C for 10 min and loaded onto a C18 column (Agilent, Santa Clara, CA, USA) using deionized water and then eluted with ethanol containing 10 mM HCl. On the day PET scans were conducted, ~3.7 MBq of ^68^Ga labelled tracer was administered to the nude mice under isoflurane anaesthesia via tail vein injection. PET scans were performed using an Inveon microPET scanner (Siemens Medical Solutions, Erlangen, Germany). Dynamic image acquisition continued for 60 min after administration. For each scan, regions of interest (ROIs) were determined using vendor software (ASI Pro 5.2.4.0) of decay-corrected whole-body coronal images. The radioactivity concentrations (accumulation) were obtained from mean pixel values within the multiple ROI volume and then converted to MBq/ml. Assuming a tissue density of 1 g/ml, these values were then divided by the administered activity to obtain an image-ROI-derived percent injected dose per gram.

### Histology and immunohistochemistry

Tumour tissues embedded in paraffin were sectioned (8 μm thickness) and then stained with hematoxylin and eosin (Beyotime Institute of Biotechnology, Jiangsu, China). For immunohistochemical staining, the sections were incubated with an anti-Ki67 antibody (Cat. #: ab15580, Abcam, Shanghai, China) at 4 °C overnight and then incubated with HRP-conjugated secondary antibodies. The sections were visualized using a DAB substrate kit (Shanghai Bio-Platform Technology Company, Shanghai, China) and an Olympus light microscope (Tokyo, Japan).

### TUNEL assay

Apoptotic cells were assessed using a terminal deoxynucleotidyl transferase dUTP nick end labelling (TUNEL) assay in paraffin-embedded tumour sections fixed on slides. The slides were stained using a TUNEL assay kit (Beyotime Institute of Biotechnology, Jiangsu, China) following the manufacturer’s instructions. Nuclei were counter-stained with hematoxylin. Staining was visualized using a Magscanner KF-PRO-120 (Konfoong Bioinformation Tech, Ningbo, China).

### Statistics

Data are reported throughout as mean ± standard deviation with significance defined as *p* < 0.05. In vivo studies were randomized, and observers didn’t know the group allocation. Statistical analyses were performed using GraphPad Prism 9.0 software with one-way ANOVA followed by Dunnett’s post-hoc test when comparing multiple independent groups. An unpaired *t*-test was used to analyse differences between two groups. Two-way ANOVA was used to analyse data from treatment and control groups with or without serum stimulation.

## Results

### Afatinib decreases the viability of Mel202, 92.1, C918 and OMM-1 cells

**Fig. 1:**
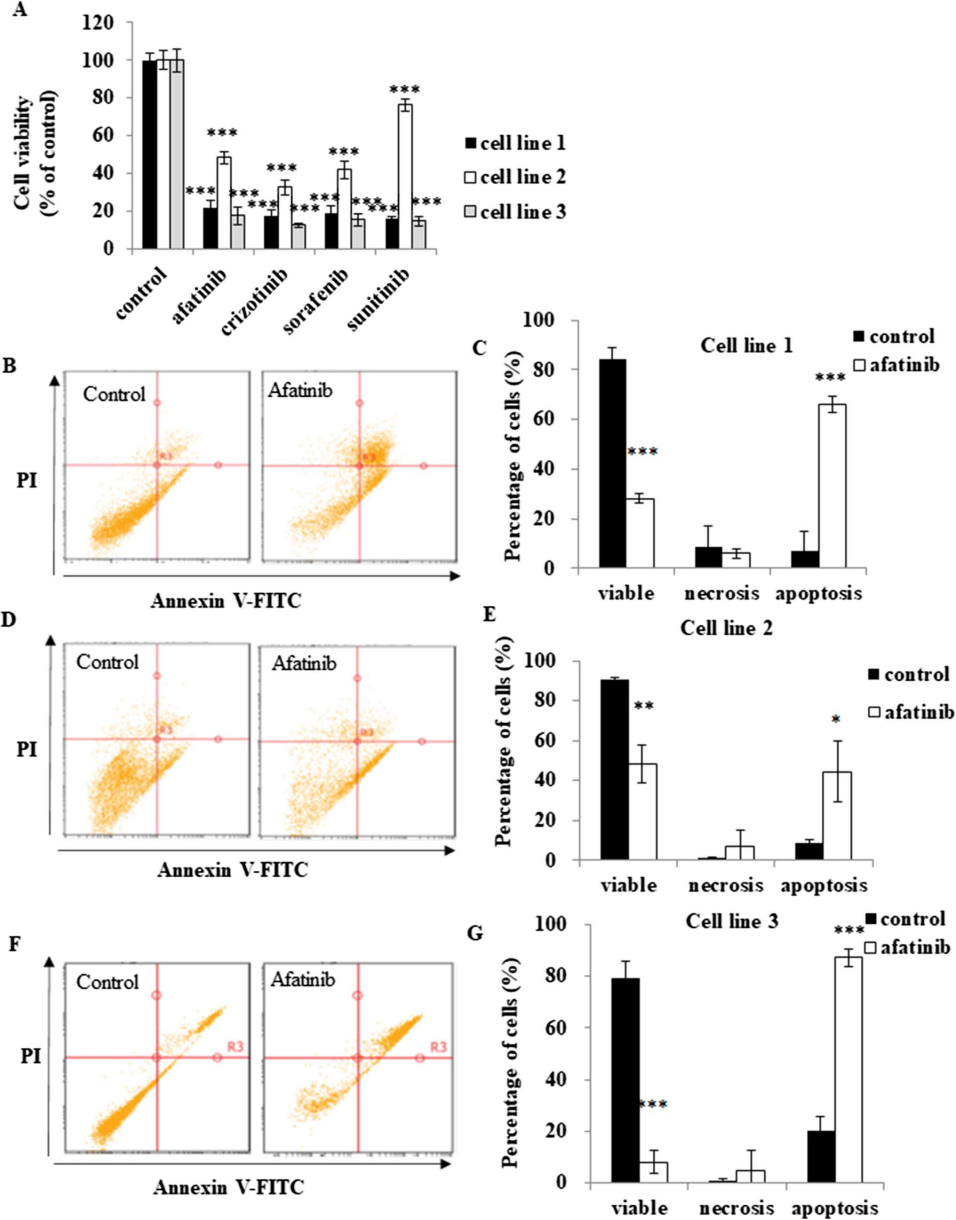
Afatinib and three other MKIs reduce cell viability and induce apoptosis in primary UM tumor-derived cell lines. UM tumor-derived cell lines were treated with or without MKIs (5 μM) for 24 h at 37 °C. Cell viability was assessed using a cytotoxicity assay (**A**). Cell death profiles in response to afatinib treatment were determined using annexin V/PI staining flow cytometry. Representative images from flow cytometry are shown in (**B**, **D** and **F**). Viable, necrotic or apoptotic cells are presented as percentages of total cells (mean ± SD) in (**C**, **E** and **G**); DMSO was used as control. Experiments were performed on three independent patient UM tumor-derived cell lines (*n* = 3 in each experiment). *p < 0.05; **p < 0.01; ***p < 0.001 vs. control by One-way ANOVA and Dunnett’s post-hoc test

In initial experiments, the capacity of 13 MKIs (10 μM, 24 h) to decrease cell viability was assessed in Mel202, 92.1, C918 and OMM-1 UM. The Mel202, 92.1 and C918 cell lines were derived from primary UM tumours, while OMM-1 is a well-established subcutis metastatic UM cell model. The concentration of 10 μM was selected in initial screening experiments because it exceeds the reported serum trough levels in patients who were treated with the MKIs tested in the present study. This was done to ensure effective cell killing across multiple UM cell lines. The most active agent across the four UM cell lines (< 20% viability remaining) was afatinib, while pelitinib was also active in Mel202 and OMM-1 cells; cediranib, foretinib, lapatinib and neratinib were most effective in OMM-1 cells (Supplementary Table 1). It was also noticed that the established EGFR inhibitor gefitinib exhibited relatively low anti-cancer activity across the four UM cells tested, which is consistent with clinical observations [18, 23]. Based on these findings, afatinib was selected for further study in the four UM cell lines and in primary UM tumour-derived cell lines. Sorafenib (a RAF/MEK/ERK and VEGFR-2/PDGFR-beta inhibitor), crizotinib (an ALK and ROS1 inhibitor) and sunitinib (a PDGFR, KIT and VEGFR inhibitor) were included in subsequent studies as controls, because these agents are currently in clinical trials in UM patients. We found that the IC_50_ values for afatinib in four cell lines that represent primary and subcutis metastatic UM were in the range 3.43–5.29 μM (Table 1). The other three agents were somewhat less potent, with the exception of sorafenib and sunitinib in OMM-1 cells (Table 1). In accord with these findings, afatinib and the other three MKIs effectively decreased the viability of patient-derived primary UM tumour cells (Fig. 1). The MKIs were more active in two of the primary cell lines while the third was somewhat less responsive (Fig. 1A). Importantly, the decrease in viability induced by afatinib was selective to UM cells, because the viability of several non-carcinoma-derived retinal cell types, including human retinal pigment epithelium cells (ARPE-19), Müller cells (MIO-M1), primary cultured melanocytes and fibroblasts were not impaired, while the other tested MKIs demonstrated mild to moderate toxicity (Supplementary Fig. 2).

**Table 1:** Cytotoxicity of afatinib, crizotinib, sorafenib and sunitinib in four human UM cell lines. Cells were treated with MKIs at concentrations between 0.01 and 50 μM (24 h, 37 °C). Cell viability was assessed using MTT cytotoxicity assays. Experiments were repeated on three occasions (*n* = 3 replicates in each experiment). Data are presented as percentage of control (mean ± SD). IC_50_ values were estimated using GraphPad Prism 9.0 software

UM cell line	IC_50_ (μM)			
	afatinib	crizotinib	sorafenib	sunitinib
Mel202	5.29 ± 1.21	7.82 ± 1.52	11.09 ± 1.88	25.85 ± 1.28
92.1	4.52 ± 1.41	13.64 ± 1.12	12.81 ± 2.26	18.39 ± 1.78
C918	3.43 ± 0.82	14.90 ± 1.09	10.93 ± 0.92	13.31 ± 1.06
OMM-1	4.47 ± 1.16	7.16 ± 1.82	4.00 ± 1.20	4.75 ± 1.71

### Afatinib induces apoptosis in Mel202, 92.1, C918 and OMM-1 cells

**Fig. 2:**
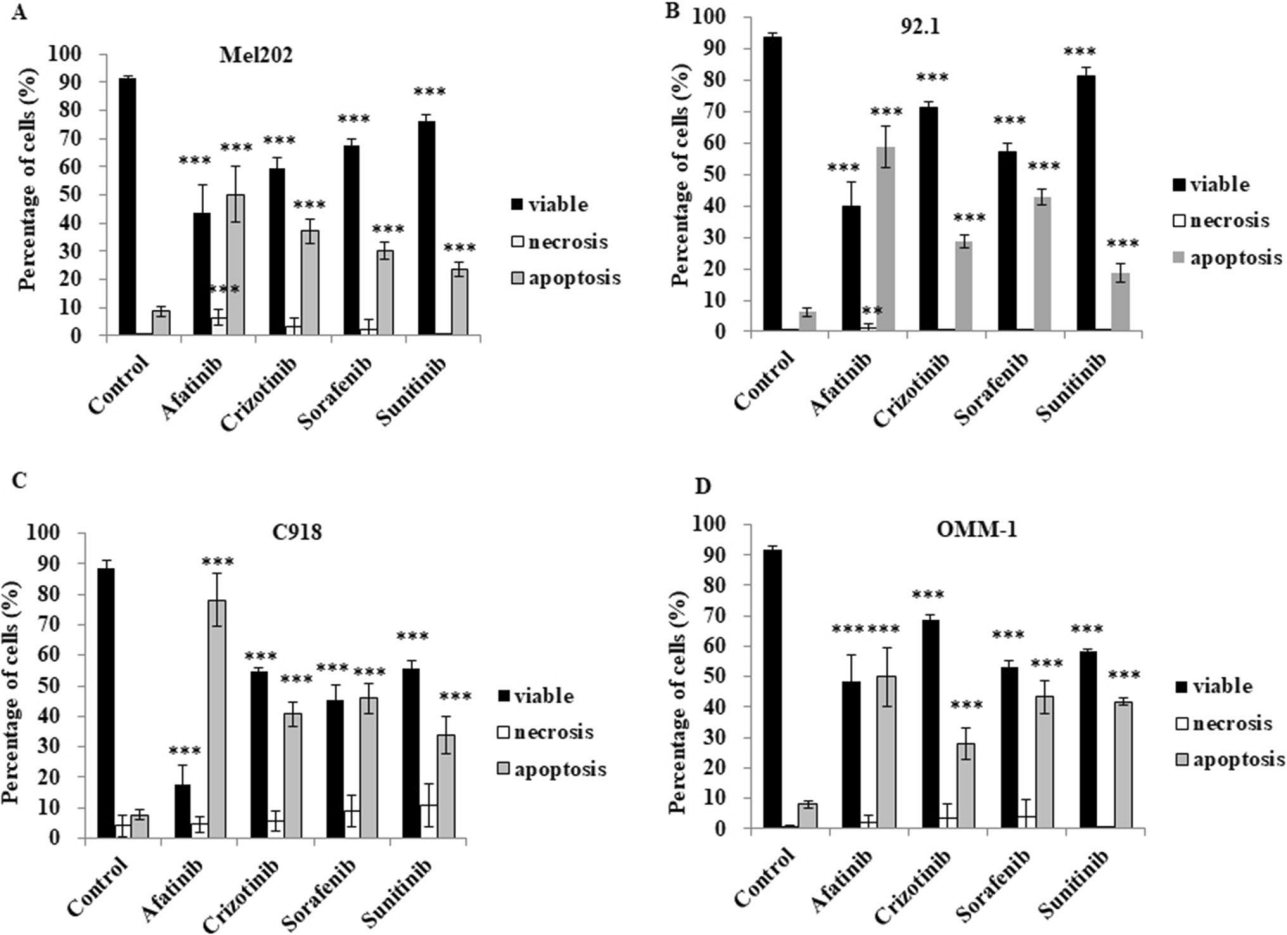
Afatinib and three other MKIs induce apoptosis in Mel202, 92.1, C918 and OMM-1 cells. Cells were treated with 5 μM MKIs for 18 h. Cell death profiles in response to MKI treatments were determined using annexin V/PI staining flow cytometry; DMSO was used as control. Cell death profiles are shown for Mel202 (**A**), 92.1 (**B**), C918 (**C**) and OMM-1 (**D**) cells. Viable, necrotic or apoptotic cells are presented as percentages of total cells (mean ± SD). Experiments were repeated on three occasions (*n* = 3 or 4 in each experiment). **p < 0.01; ***p < 0.001 vs. control by One-way ANOVA and Dunnett’s post-hoc test

The decreases in UM cell viability induced by afatinib were evaluated further in annexin V/PI-stained cells using flow cytometry. An afatinib concentration of 5 μM was selected based on IC_50_ values estimated in the four UM cell lines (Table 1). We found that apoptosis was the primary cause of death in MKI-treated UM cells (Fig. 2). Afatinib treatment increased the proportion of apoptotic cells 5.79–10.20-fold to that of the control, while the increases induced by the other three agents were somewhat less pronounced (to 3.50–5.32-fold to that of the control by crizotinib, to 3.50–6.84-fold to that of the control by sorafenib and to 2.73–5.26-fold to that of the control for sunitinib; Supplementary Table 2). Consistent with these findings, we found that afatinib treatment also activated apoptosis in primary tumor-derived cells obtained from three UM patients (Fig. 1B-G).

**Fig. 3:**
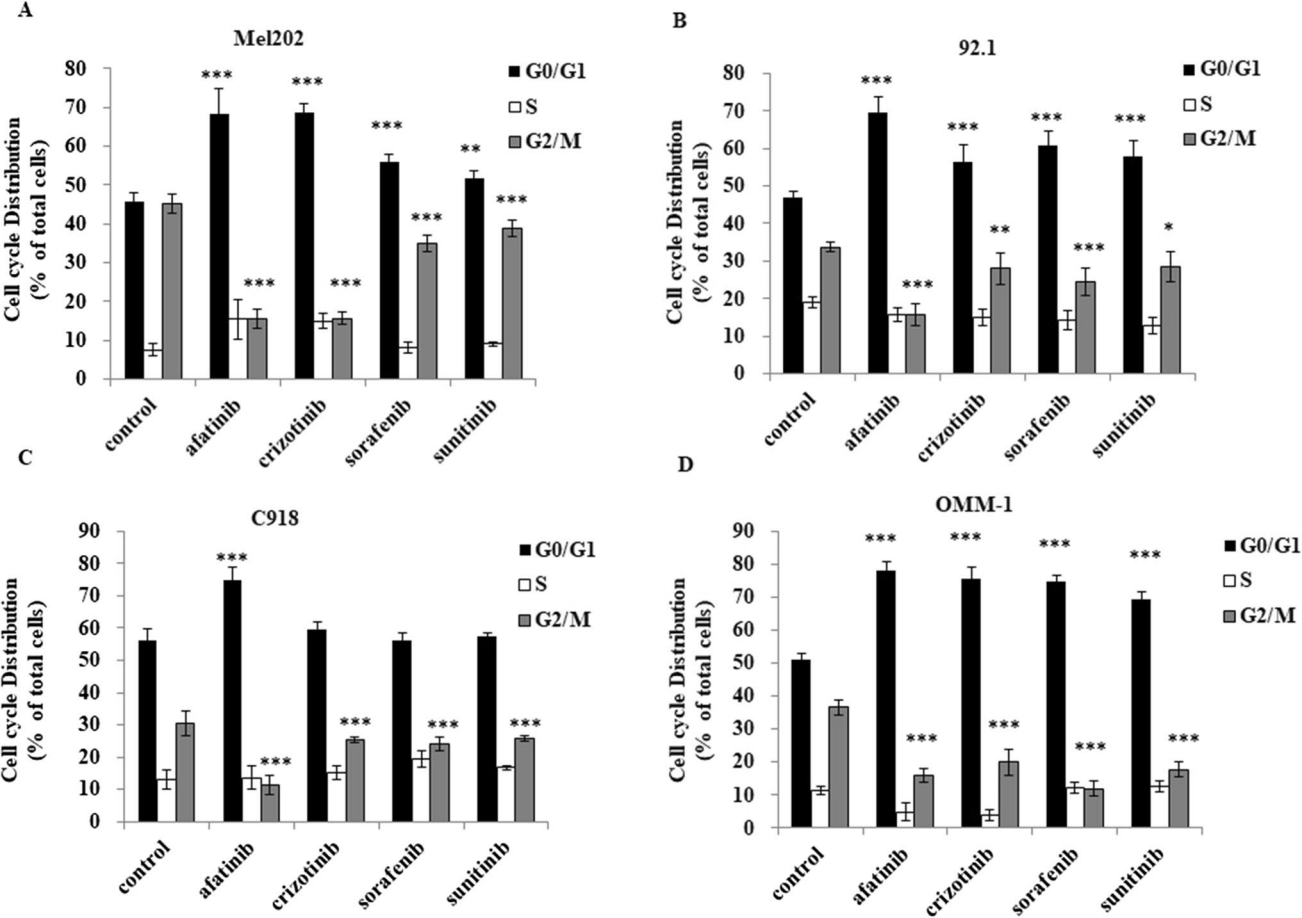
Afatinib and three other MKIs induce cell cycle arrest in Mel202, 92.1, C918 and OMM-1 cells. Cells were treated with 5 μM MKIs for 18 h. Cell cycle profiles were determined by PI staining flow cytometry. DMSO was used as control. Cell cycle distributions are shown for Mel202 (**A**), 92.1 (**B**), C918 (**C**) and OMM-1 (**D**) cells. Distributions of cells in G0/G1, S or G2/M phases are presented as percentages of total cells (mean ± SD). Experiments were repeated on three occasions (*n* = 3 in each experiment). *p < 0.05; **p < 0.01; ***p < 0.001 vs. control by One-way ANOVA and Dunnett’s post-hoc test

Further cell cycle analysis revealed that afatinib arrested UM cells in the G0/G1 phase and decreased entry into the G2/M phase (Fig. 3). In OMM-1 cells G0/G1 accumulation and G2/M suppression was extensive and all of the MKIs were found to be similarly active. Taken together, the cell death and cell cycle analyses indicate that afatinib is highly effective in inducing apoptosis and enhancing cell cycle arrest in UM cell lines.

### Afatinib treatment decreases cell migration and promotes reproductive cell death in Mel202, 92.1, C918 and OMM.1 cells

**Fig. 4:**
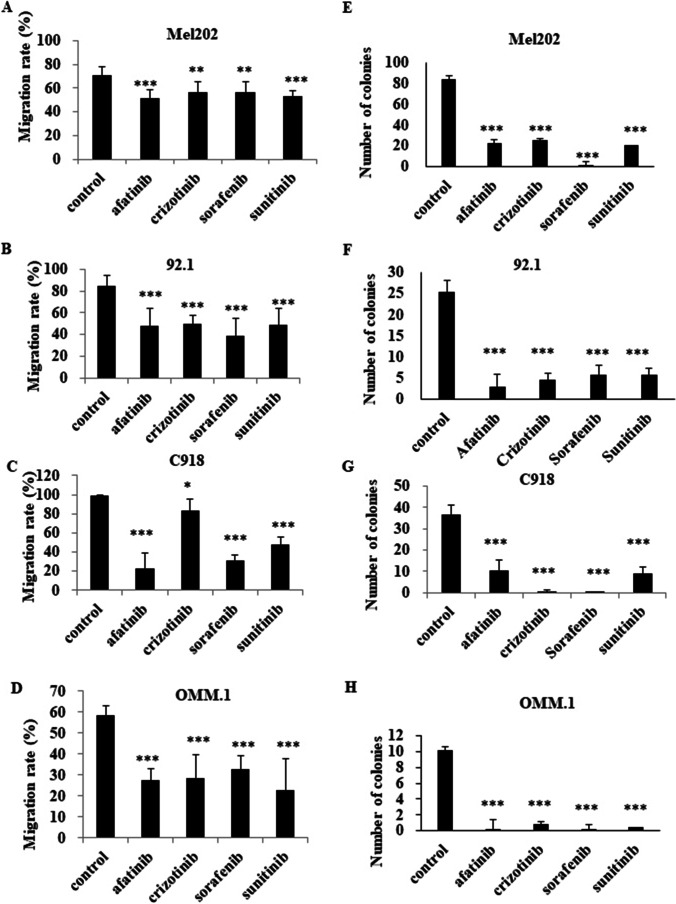
Afatinib and other three MKIs reduce cell migration and promote reproductive cell death in Mel202, 92.1, C918 and OMM-1 cells. A cell migration assay was performed on UM cells treated with 5 μM MKIs for 24 h. Images were taken at the start of the experiment (0 h) and 24 hours later (24 h). The means ± SD of migration rates are shown for Mel202 (**A**), 92.1 (**B**), C918 (**C**) and OMM-1 (**D**) cells. Experiments were repeated on three occasions (*n* = 2 estimates in each experiment). Colony formation assays were performed as described in Materials and methods. Means ± SD of colony numbers are shown for Mel202 (**E**), 92.1 (**F**), C918 (**G**) and OMM-1 (**H**) cells. Experiments were repeated on three occasions (*n* = 4 estimates in each experiment). *p < 0.05; **p < 0.01; ***p < 0.001 vs. control by One-way ANOVA and Dunnett’s post-hoc test

We tested the capacity of the four MKIs to decrease UM cell migration using scratch wound-healing assays. We found that afatinib significantly decreased migration rates, especially in C918 cells, while the effects in Mel202, 92.1 and OMM.1 cells were somewhat less pronounced (Fig. 4A-D). Colony formation assays were performed to subsequently evaluate the capacity of these four MKIs to decrease the viability of Mel202, 92.1, C918 and OMM.1 cells. We found that the four MKIs (5 μM) also impaired the colony growth of UM cells using clonogenic assays (Fig. 4E-H), consistent with the induction of reproductive cell death.

### STAT1-regulated apoptotic pathways contribute to the anti-cancer action of afatinib in UM cells

**Fig. 5:**
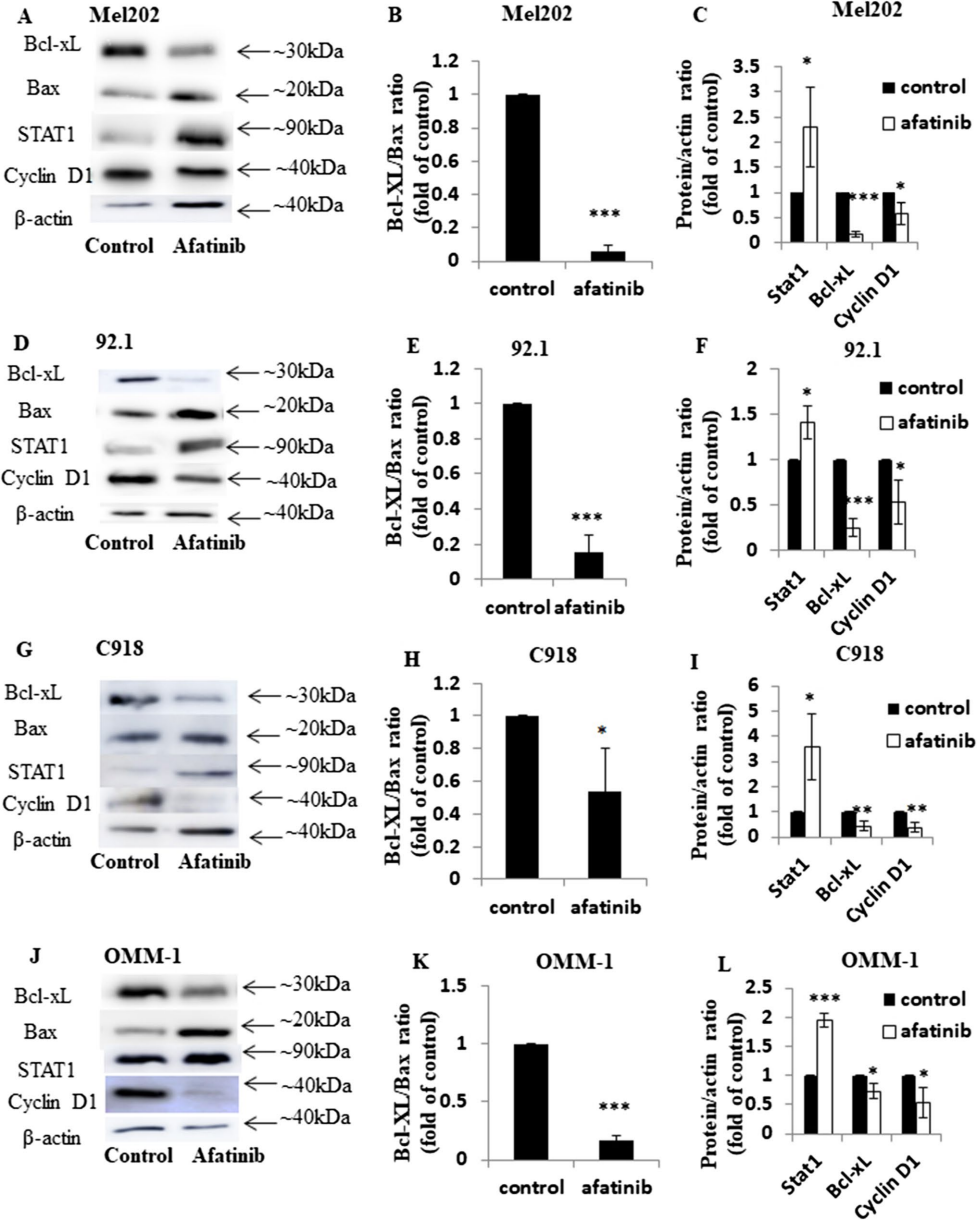
Afatinib induces apoptosis by regulating STAT1, Bcl-xL and cyclin D1 in Mel202, 92.1, C918 and OMM-1 cells. Expression of Bcl-xL, Bax, STAT1 and cyclin D1 was assessed using Western blotting in Mel202, 92.1, C918 and OMM-1 cells after treatment with afatinib (5 μM) for 24 h at 37 °C; β-actin was used as loading control. Representative images are shown for Mel202 (**A**), 92.1 (**D**), C918 (**G**) and OMM-1 (**J**) cells. Densitometry analysis of each protein was conducted. Ratios of Bcl-xL to Bax expression upon treatment with afatinib or vehicle control are shown for Mel202 (**B**), 92.1 (**E**), C918 (**H**) and OMM-1 (**K**) cells. The relative expression of STAT1, Bcl-xL and cyclin D1 compared to β-actin is shown for Mel202 (**C**), 92.1 (**F**), C918 (**I**) and OMM-1 (**L**) cells after treatment with afatinib or vehicle control. Data are presented as fold of control (mean ± SD). Experiments were repeated on three occasions. *p < 0.05; **p < 0.01; ***p < 0.001 vs. control by unpaired t-test

The capacity of afatinib to modulate the expression of important Bcl-2 proteins - Bax (pro-apoptotic) and Bcl-xL (anti-apoptotic) - that regulate apoptosis, was assessed in UM cells (Fig. 5). We found that afatinib increased the expression of Bax and decreased that of Bcl-xL leading to Bcl-xL/Bax ratios that were decreased to <0.2-fold compared to that in control in Mel202, 92.1 and OMM-1 cells (*p* < 0.001; Fig. 5B, E and K) and to ~0.5 fold compared to that in control in C918 cells (*p* < 0.05; Fig. 5H). Because afatinib induced cell cycle arrest, we also evaluated the expression of the important mediator cyclin D1 in afatinib-treated UM cells and found that it was decreased to 0.4–0.6-fold compared to that in control cells (Fig. 5).

STAT1 acts as a tumour suppressor in a range of cancer types and is associated with the expression of Bcl-xL and cyclin D1 [37]. We found that the expression of STAT1 was significantly increased in the UM cell lines to 1.4–3.5-fold compared to that in controls by afatinib treatment (Fig. 5C, F, I and L). Overall, we found that afatinib induced apoptosis in UM cells in a STAT1- and Bcl-xL/cyclin D1-dependent manner.

### HER2 signalling may be involved in the anti-cancer effect of afatinib in UM cells

**Fig. 6:**
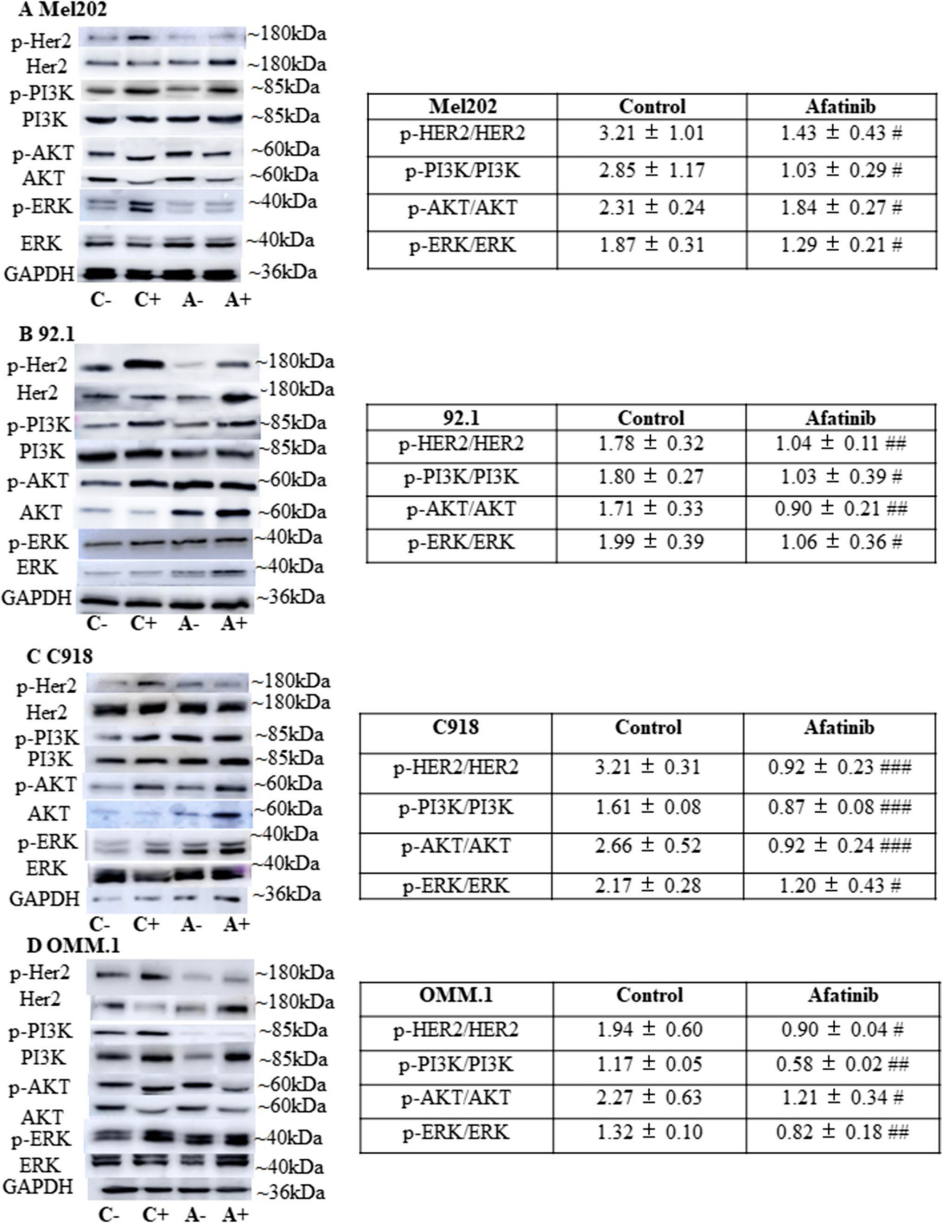
Afatinib exerts its anti-cancer actions by targeting the HER2, AKT, ERK and PI3K signalling cascades in UM cells. Serum was removed from Mel202, 92.1, C918 and OMM-1 cells and 24 h later, cells were treated with 20% FBS or medium alone for 10 min at 37 °C. Subsequently, cells were treated with afatinib or vehicle (serum-free medium) for 1 h at 37 °C prior to the preparation of total cell lysates. The expression of HER2, PI3K/AKT and PI3K signalling proteins was analysed by Western blotting. Representative images of p-HER2, HER2, p-AKT, AKT, p-PI3K, PI3K, p-ERK and ERK are shown for Mel202 (**A**), 92.1 (**B**), C918 (**C**) and OMM-1 (**D**) cells; GAPDH was used as loading control. Densitometry analysis of protein expression was preformed. Ratios of p-HER2/HER2, p-AKT/AKT, p-PI3K/PI3K and p-ERK/ERK with serum stimulation are presented as fold of those without serum stimulation (tables at right). Data are presented as fold of control (mean ± SD). Experiments were repeated on three occasions. ^#^*p* < 0.05; ^##^*p* < 0.01; ^###^*p* < 0.001 vs. control by Two-way ANOVA. Note: C-: control without serum stimulation; C+: control with serum stimulation; A-: afatinib treatment without serum stimulation; A+: afatinib treatment with serum stimulation

Afatinib has been reported to act as a dual inhibitor of EGFR and HER2 in non-small cell lung cancer and other cancer cells [38]. In this study, we assessed the involvement of EGFR and HER2 in the anti-cancer actions of afatinib in UM cells. We tested the activation of both receptors in response to acute stimulation by serum growth factors. Previously, it has been reported that EGFR is only expressed in a small proportion of UM tumours and immortalised cell lines [16–20, 22]. Consistent with these reports, we failed to detect EGFR expression in Mel202, 92.1, C918 and OMM-1 cells and found that phospho-EGFR expression did not increase following serum stimulation (Supplementary Fig. 3). Therefore, it is unlikely that EGFR signalling mediates the anti-UM effect of afatinib in UM cells. In contrast, we observed HER2 expression in all four UM cell lines and, in addition, that serum stimulation (20% FBS, 10 min) produced rapid activation of HER2, as indicated by increased p-HER2 expression (Fig. 6). Moreover, signalling pathways downstream from HER2 - most notably the ERK, PI3K and AKT pathways - were also found to be activated by serum (Fig. 6). Subsequent treatment with afatinib (1 h) markedly attenuated the serum-induced activation of HER2 and its downstream signalling in UM cells (Fig. 6). Taken together, we found that acute inhibition of HER2, PI3K, AKT and ERK signalling occurred after the administration of afatinib and preceded the loss of UM cell viability.

### Afatinib shows anti-tumour activity in a UM cell xenograft model

**Fig. 7:**
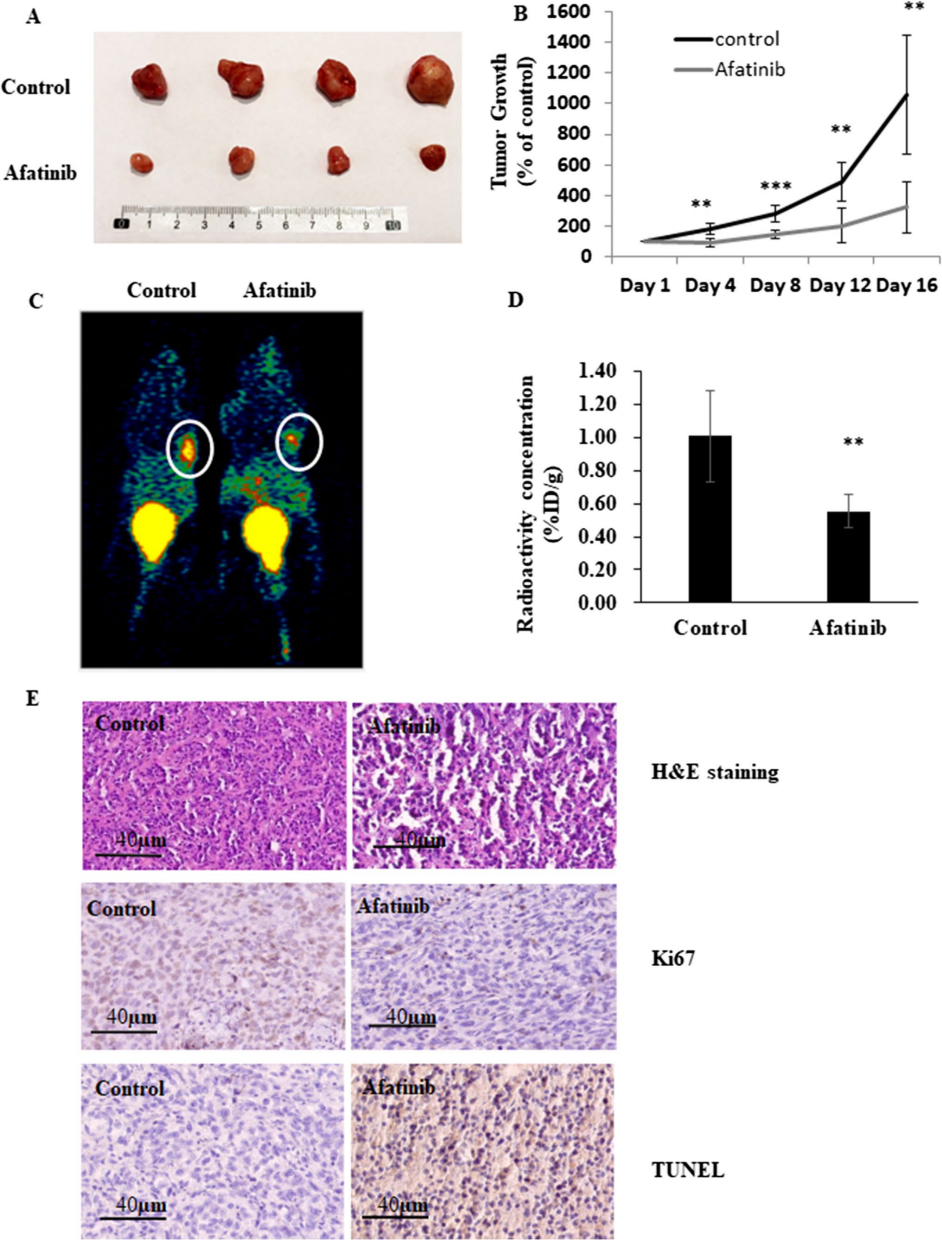
Afatinib inhibits tumour growth in a UM cell xenograft model. BALB/c nude mice were inoculated with C918 cells and maintained for 14 days. Mice were administered afatinib (15 mg/kg per day, *n* = 10) or vehicle (*n* = 12) on day 15 via intraperitoneal injection; treatments were continued for another 16 days. Tumor volumes and body weights of mice were measured every 4 days. At the end of the experiment mice were either sacrificed for the harvesting of tumor samples or were subjected to whole body PET scans (*n* = 5 or 6 mice in each arm). Representative images of tumours are shown in panel (**A**). Tumor growth curves are shown in panel (**B**). Data are shown as percentages of the tumor size on day 15 (mean ± SD; *n* = 5 or 6 per group). **p < 0.01; ***p < 0.001 vs. control by unpaired t-test. Representative PET scan images are shown in panels (**C**) and (**D**). Positron-emission intensity of tumours is presented as radioactivity vs. weight (% ID/g; mean ± SD, *n* = 5 or 6 per group). **p < 0.01 vs. control by unpaired t-test. Representative images of staining analyses of tumour sections are shown in panel (**E**). Paraffin-embedded tissue cryostats from control and afatinib-treated mice were subjected to hematoxylin and eosin staining (upper), anti-Ki67 (middle or TUNEL assays (bottom)

The activity of afatinib was further evaluated in an in vivo C918 UM cell xenograft mouse model. We found that afatinib treatment (15 mg/kg daily for 16 days) markedly decreased the final weights of C918-derived UM tumours in mice (Fig. 7A) and strongly inhibited tumour growth (Fig. 7B). Using PET scan analysis, we found that the tumour volumes after afatinib treatment were decreased to ~55% of the controls (*p* < 0.01; Fig. 7C and D).

Subsequently, we performed immunohistochemical staining of the tumour tissues harvested from the xenografted mice (Fig. 7E). We found that there was a pronounced decrease in staining for the tumor proliferation marker Ki67 in the UM tumours following afatinib treatment. Increases in TUNEL staining indicated that apoptosis was activated in tumours isolated from afatinib-treated mice. These findings indicate that afatinib effectively induces apoptosis and inhibits proliferation in UM tumours in vivo.

## Discussion

UM has a poor prognosis and, currently, there are no effective treatment options. MKI drugs have revolutionised the treatment of many cancer types by targeting kinases that drive important tumorigenic mechanisms such as proliferation, survival, motility and angiogenesis. Gefitinib, crizotinib and afatinib are kinase inhibitors that are used to treat tumours that exhibit *EGFR* mutations, *ALK* fusions and *ERBB2/HER2*-amplification, respectively [39–41]. Other inhibitors like sorafenib target multiple tumorigenic kinases [42]. MKIs that are known to be clinically useful for certain cancers have; however, yielded disappointing results in clinical trials for UM [14, 15].

The EGFR family encompasses four ErbB members (ErbB1–4) that form homo- and hetero-dimers [25]. Apart from HER2, the receptors contain an extracellular domain with leucine-rich regions that can bind growth factors [25]. EGFR family proteins have been widely studied as anti-cancer targets [43]. Initial studies suggested that EGFR was expressed in human UM cell lines and tumours and that the expression correlated with tumorigenic activities, including proliferation and metastatic potential [44, 45]. It has been reported that 14 of 48 primary UMs and 3 of 14 UM cell lines over-expressed EGFR and that EGFR over-expressing tumours, but not EGFR negative tumours, showed an activated EGF-signature [18]. In another series of 21 primary UMs tested, EGFR was detected in 6 of them and was found to correlate with metastatic disease [17]. In yet another study EGFR was found to be expressed in 8 of 40 tumours and to correlate with mitotic activity [16]. Other studies have, however, questioned the significance of EGFR in UM progression. In a study encompassing 60 UM tumours of varying aggressiveness, EGFR expression was found to be positive in 13 and heterogeneous in 5 [20]. No correlation was observed between EGFR expression and tumorigenic activity. Scholes et al. [22] and Mallikarjuna et al. [20] failed to observe any associations between EGFR expression and tumorigenic or metastatic capacities [16]. Moreover, gefitinib treatment yielded only limited benefits in a phase II study in 50 patients with UM or metastatic cutaneous melanomas. Only one of 6 patients with UM exhibited a response with a progression-free survival period of 9.7 months [23]. In the present study, selective EGFR inhibitors (erlotinib, gefitinib and vandetanib) were found to be relatively ineffective in decreasing UM cell viability and none of the UM cell lines used in the present study expressed EGFR. Therefore, EGFR is unlikely to be a significant target of afatinib in UM. In addition, two of the four UM cell lines that were used in the present study did not express HER4 (data not shown), suggesting HER4 is also unlikely a significant target for afatinib.

Consistent with findings in the present study, it has been reported that HER2 is expressed in UM cells [46]. Forsberg et al. presented confirmatory evidence that HER2 protein in xenograft models of UM is detectable by immunohistochemical staining [47]. We found that MKIs that inhibit HER2 were more effective in decreasing UM cell viability. Afatinib was more potent in decreasing UM cell viability than three other MKIs (crizotinib, sorafenib and sunitinib) that are currently in clinical trials. Afatinib strongly activated apoptosis and cell cycle arrest in the four UM cell lines tested in this study, which was corroborated in UM patient tumour-derived cell lines. Interestingly, afatinib also demonstrated a significant inhibitory effect on UM cell migration and promoted reproductive cell death, which indicates its clinical potential in the inhibition of UM metastasis. Our data also showed anti-cancer activity of afatinib in a UM xenograft model. Afatinib markedly inhibited tumour growth and suppressed tumour progression, which suggests that the drug may have an in vivo therapeutic potential.

**Fig. 8:**
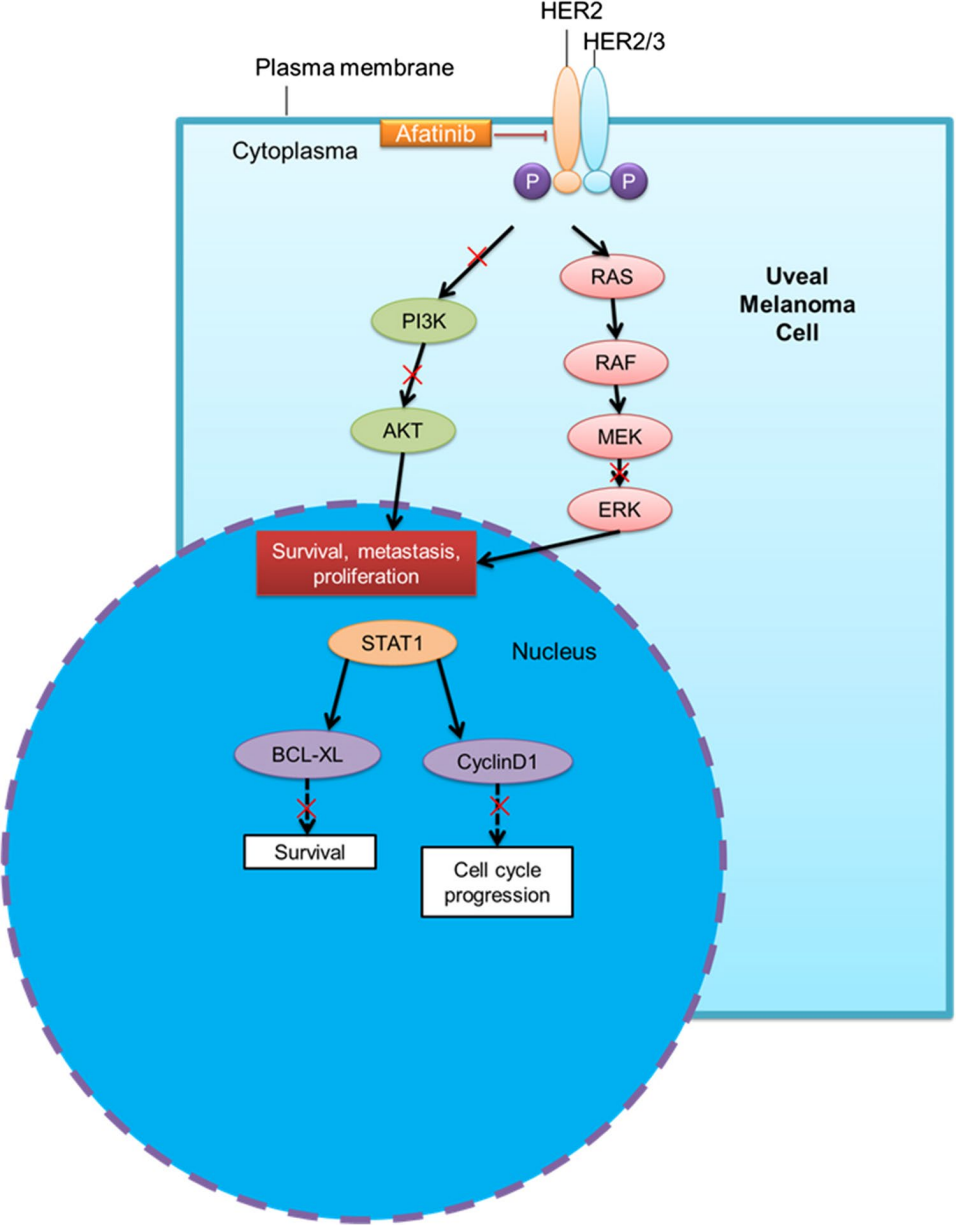
Schematic summary of the anti-cancer mode of action of afatinib in UM cells. Afatinib inhibits HER2 to exert its anti-UM effect, which then leads to downregulation of the downstream PI3K, AKT and ERK pathways. These early events activate UM cell apoptosis and decrease UM cell survival and progression by inducing STAT1 and downregulating Bcl-xL and cyclin D1

Afatinib-induced apoptosis is associated with decreased expression of the anti-apoptotic Bcl-xL protein, the cell cycle progression regulator cyclin D1, and a decrease in the Bcl-xL/BAX ratio in UM cell lines. Afatinib-driven cellular apoptosis was also accompanied by an elevated expression of STAT1. STAT1 plays an important role in cell survival, viability and responses to pathogens. STAT1 induces cell cycle arrest in response to interferon-γ by interacting with D-type cyclins and cyclin-dependent kinase-4 in fibrosarcoma cells [48]. STAT1 also inhibits the transcription of the anti-apoptotic Bcl-2 and Bcl-xL proteins that promote mitochondrial integrity [49, 50]. STAT1 expression has been reported to be increased in EGFR-positive and HER2-positive breast cancer patients, and relapse-free survival was found to be decreased in high-risk breast cancer patients [26]. STAT1 expression is transcriptionally upregulated by HER2 in breast cancer cells [51]. In stably transfected cell lines that overexpress HER2, 462 proteins were detected using the SILAC (stable isotope labelling with amino acids in cell culture) method, 198 of them showing increased tyrosine phosphorylation and 81 showing decreased tyrosine phosphorylation [27]. These phosphoproteins included a number of HER2 and EGFR signalling intermediates such as STAT1 [27]. STAT1 expression has also been found to correlate with a favorable prognosis in several cancer types, including colorectal [52, 53], hepatocellular [54] and esophageal [55] cancers, and metastatic melanomas [56]. Concomitant deletion of STAT1 and overexpression of the ErbB2/neu oncogene in mammary epithelial cells accelerated mammary tumorigenesis [57, 58]. Consistent with literature, in the current study afatinib was found to increase STAT1 expression and to decrease the expression of downstream cyclin D1 and Bcl-xL. Thus, the anti-UM effects of afatinib are likely mediated through STAT1 upregulation that subsequently leads to cell cycle arrest and apoptosis (Fig. 8).

We confirmed HER2 activation upon acute afatinib treatment in all the four UM cell lines tested, which suggests that HER2 is likely the molecular target of afatinib and, thus, may be a viable anti-cancer target in UM. Unlike the EGFR inhibitors erlotinib and gefitinib that act in a reversible fashion, the HER2 inhibitors afatinib, pelitinib and neratinib also include an irreversible component in their inhibitory mechanism [25]. These agents bind to the ATP pocket of the receptor and their bulky extra-aromatic groups are oriented toward the kinase domain of HER2 [59, 60]. Moreover, three other HER2 inhibitors that were assessed in an initial screening (lapatinib, pelitinib and neratinib) showed more effective anti-UM activity than other MKIs that did not target HER2.

Several signalling cascades, including the AKT, PI3K and ERK pathways, have been shown to be activated by HER2 [59, 60]. These were also evaluated in afatinib-treated UM cells. We found that afatinib markedly attenuated the activation of HER2 and the downstream signalling AKT, ERK and PI3K-linked cascades in UM cells. Thus, the inhibitory effect of afatinib on HER2, PI3K, AKT and ERK signalling could be an early event following afatinib treatment resulting in a loss of UM cell viability (Fig. 8).

Afatinib is clinically approved to treat non-small cell lung carcinoma (NSCLC) and head and neck squamous carcinoma [61–64]. A phase II trial of afatinib monotherapy showed some promise in patients with HER2-positive esophagogastric cancers that were refractory to the anti-HER2 monoclonal antibody trastuzumab [65]. Our study is the first to show the clinical potential of afatinib in the treatment of UM and the prevention of metastasis. Importantly, our data show that afatinib is highly selective to UM tumour cells while minimally altering the viability of normal retinal cells, melanocytes and fibroblasts. In contrast, the other three MKIs tested were somewhat toxic to non-carcinoma retinal cell types. Therefore, afatinib may have greater efficacy and lower toxicity in the treatment of UM. The C_max_ of afatinib is ~0.16 μM after administration of multiple daily oral doses of 50 mg in patients [66]. Brain penetration of afatinib has been demonstrated in in vivo models, although it might be somewhat lower than other EGFR inhibitors [67, 68]. The desired plasma concentration of afatinib may not be readily achievable in the treatment of UM by oral route. However, intraocular injection, or other local administration routes that are used commonly for the treatment of eye diseases, may enable higher local concentrations to be achieved. In addition, advances in novel drug formulations may allow improved delivery of afatinib for the treatment of UM. Future research into these areas is now warranted, but is beyond the scope of the current study.

In summary, we found that afatinib has potent anti-cancer properties in in vitro, ex vivo and in vivo UM models. HER2 signalling has emerged as a likely molecular target that activates apoptosis upon afatinib treatment in UM cells. Afatinib also has the advantage of preventing UM cell migration and enhancing reproductive cell death, which may contribute to suppressing UM metastasis. Together, our data indicate that afatinib may serve as a novel candidate drug with an improved therapeutic effect and selectivity in treating UM by targeting HER2.

## Supplementary Information

ESM 1 (DOCX 721 kb)

## Data Availability

The datasets generated and/or analysed during the current study are available from the corresponding author upon reasonable request.

## Abbreviations

ADAM: A Disintegrin and Metalloproteinase
ASAP1: Arf-GAP with SH3 Domain
BAP1: BRCA 1-associated protein-1
BAX: Bcl-2-associated X Protein
BCG: Bacillus calmette-guerin
BRD: Bromodomain
c-Met: Tyrosine-Protein Kinase Met
CD47: Cluster of Differentiation 47
CNKSR3: Connector Enhancer of Kinase Suppressor of Ras 3 on Chromosome 6
CR: Complete Response
CTL: Cytolytic T lymphocyte
CTLA-4: Cytotoxic T lymphocyte antigen 4
CYSLTR2: Cysteinyl leukotriene receptor-2
EGFR: Epidermal Growth Factor Receptor
EIF1AX: Eukaryotic Translation Initiation Factor 1A X-Linked
FAK: Focal Adhesion Kinase
GNA11: Guanine Nucleotide-Binding Protein Gq Subunit alpha-11
GNAQ: Guanine Nucleotide-Binding Protein Gq Subunit alpha
HDAC: Histone deacetylase
HDACi: HDAC inhibitor
HER-2: Human Epidermal Growth Factor Receptor-2
HGF: Hepatocyte Growth Factor
IGF-1: Insulin-like growth factor 1
IGFR: Insulin-like growth factor receptor
KI: Kinase Inhibitor
LZTS1: Leucine Zipper Putative Tumour Suppressor-1 on Chromosome 1
MAPK: Mitogen-Activated Protein Kinase
MHC: Major Histocompatibility Complex
miRNA: Micro RNA
MMP: Matrix Metalloproteinase
NFκB: Nuclear factor-kappa B
NF1: Neurofibromin 1
NK: Natural Killer
NRAS: Neuroblastoma RAS viral oncogene homolog
OS: Overall survival
PD-1: Programmed cell death 1
PD-L1: Programmed death-ligand 1
PFS: Progression-free survival
PI3K: Phosphatidyinositol 3-Kinase
PKC: Protein kinase C
PLCB4: Phospholipase C-β4
PR: Partial Response
PTP4A3: Protein Tyrosine Phosphatase 4A3 on Chromosome 8
RTK: Receptor Tyrosine Kinase
SD: Stable Disease
SF3B1: Splicing factor 3b subunit 1
TCEB1: Transcription Elongation Factor Polypeptide 1
TGF: Transforming Growth Factor
TKI: Tyrosine kinase inhibitor
TNF-α: Tumour Necrosis Factor α
UM: Uveal melanoma
VEGF: Vascular endothelial growth factor
VEGFR: Vascular endothelial growth factor receptor
