# Supplementary material for: The multi-kinase inhibitor afatinib serves as a novel candidate for the treatment of human uveal melanoma
Source: Cell Oncol (Dordr). 2022 Jul 4;45(4):601–19. doi: 10.1007/s13402-022-00686-5 (PMC9424141; doi:10.1007/s13402-022-00686-5)
Supplement: Supplementary file 1 — (DOCX 721 kb) [file 13402_2022_686_MOESM1_ESM.docx]

| MKI | Molecular Target | Viability (% of control) | | | |
| --- | --- | --- | --- | --- | --- |
|  |  | Mel202 | 92.1 | C918 | OMM-1 |
| Control |  | 100.00 ± 6.07 | 100.00 ± 3.08 | 100.00 ± 5.58 | 100.00 ± 2.5 |
| **Afatinib** | **EGFR, HER2 and HER4 inhibitor** | **18.25 ± 3.25 ***** | **19.76 ± 2.11 ***** | **15.82 ± 0.73 ***** | **16.99 ± 1.32 ***** |
| Bosutinib | BCR-ABL and src tyrosine kinase inhibitor | 38.24 ± 2.98 ******* | 53.65 ± 2.77 ******* | 28.47 ± 1.68 ******* | 17.80 ± 0.84 ******* |
| Cediranib | VEGFR inhibitor | 21.24 ± 2.68 ******* | 25.71 ± 1.57 ******* | 12.87 ± 0.57 ******* | 6.21 ±1.24 ******* |
| Crizotinib | ALK and ROS1 inhibitor | 36.84 ± 6.84 ******* | 44.23 ± 3.81 ******* | 59.80 ± 2.40 ******* | 33.64 ± 3.89 ******* |
| Erlotinib | EGFR inhibitor | 72.40 ± 4.30 ******* | 63.63 ± 3.17 ******* | 12.58 ± 0.55 ******* | 39.50 ± 2.59 ******* |
| Foretinib | MET and VEGFR inhibitor | 8.51 ± 3.21 ******* | 37.45 ± 2.19 ******* | 63.41 ± 2.41 ******* | 3.3 ± 0.12 ******* |
| Gefitinib | EGFR inhibitor | 57.79 ± 10.28 ******* | 59.57 ± 3.27 ******* | 65.60 ± 2.21 ******* | 32.60 ± 2.15 ******* |
| Lapatinib | EGFR and HER2 inhibitor | 16.28 ± 3.67 ******* | 25.32 ± 1.53 ******* | 32.53 ± 0.99 ******* | 7.31 ± 0.80 ******* |
| Neratinib | HER2 inhibitor | 33.05 ± 2.43 ******* | 48.36 ±2.00 ******* | 14.47 ± 0.85 ******* | 4.61 ± 0.65 ******* |
| Pelitinib | EGFR, HER2 and HER4 inhibitor | 2.03 ± 0.07 ******* | 45.20 ± 6.94 ******* | 14.69 ± 0.65 ******* | 2.69 ± 0.16 ******* |
| Sorafenib | RAF/MEK/ERK and VEGFR-2/PDGFR-beta inhibitor | 51.38 ± 2.51 ******* | 52.08 ± 2.00 ******* | 57.13 ± 2.87 ******* | 17.75 ± 1.74 ******* |
| Sunitinib | PDGFR, KIT and VEGFR inhibitor | 71.01 ± 6.56 ******* | 63.95 ± 6.51 ******* | 59.06 ± 3.49 ******* | 32.90 ± 2.39 ******* |
| Vandetanib | VEGFR, EGFR, and the RET-tyrosine kinase inhibitor | 78.60 ± 17.27 ******* | 70.90 ± 2.61 ******* | 25.86 ± 1.12 ******* | 25.68 ± 2.43 ******* |

Supplementary Table 1. Anti-cancer effect screening of MKIs in the three UM cell lines by MTT assays.

Cells were pretreated with 10 μM of each MKI for 24 hours at 37°C. Cell viability was measured with cytotoxic assay. 0.1% DMSO in medium was used as control. Experiments were done in triplicates and repeated on three occasions. Data are presented as percentage of control (mean ± SD). ***, p<0.001 vs. control by One-way ANOVA and Dunnett’s post-hoc test.

| Treatment | Viable  (% of total cells) | Necrosis  (% of total cells) | Apoptosis  (% of total cells) | Apoptotic cells  (Fold of MKI treated group vs. control group) |
| --- | --- | --- | --- | --- |
| **MEL202 cells** | | | | |
| Control | 91.43 ± 1.01 | 0.21 ± 0.22 | 8.66 ± 1.68 | 1.00 |
| Afatinib | 43.42 ± 10.00 *** | 6.45 ± 2.63 *** | 50.15 ± 9.98 *** | 5.79 |
| Crizotinib | 59.38 ± 4.00 *** | 3.08 ± 3.15 | 37.21 ± 4.23 *** | 4.30 |
| Sorafenib | 67.36 ± 2.69 *** | 2.35 ± 3.62 | 30.28 ± 3.18 *** | 3.50 |
| Sunitinib | 76.34 ± 2.40  *** | 0.06 ± 0.06 | 23.62 ± 2.36  *** | 2.73 |
| **92.1 cells** | | | | |
| Control | 93.74 ± 1.35 | 0.01 ± 0.01 | 6.25 ± 1.35 | 1.00 |
| Afatinib | 40.07 ± 7.65 *** | 1.24 ± 1.44  *** | 58.69 ± 6.72 *** | 9.39 |
| Crizotinib | 71.22 ± 1.91 *** | 0.04 ± 0.04 | 28.74 ± 1.91  *** | 4.60 |
| Sorafenib | 57.18 ± 2.58 *** | 0.04 ± 0.03 | 42.78 ± 2.57 *** | 6.84 |
| Sunitinib | 81.19 ± 2.91 *** | 0.04 ± 0.07 | 18.77 ± 2.87 *** | 3.00 |
| **C918 cells** | | | | |
| Control | 88.40 ± 2.62 | 3.98 ± 3.67 | 7.66 ± 1.73 | 1.00 |
| Afatinib | 17.49 ± 6.60 *** | 4.38 ± 2.56 | 78.14 ± 8.62 *** | 10.20 |
| Crizotinib | 54.87 ± 1.12 *** | 5.52 ± 3.50 | 40.72 ± 4.05 *** | 5.32 |
| Sorafenib | 45.34 ± 4.87 *** | 8.86 ± 4.99 | 45.81 ± 5.05 *** | 5.98 |
| Sunitinib | 55.73 ± 2.71  ** | 10.58 ± 7.12  * | 33.68 ± 6.08  *** | 4.40 |
| **OMM-1 cells** | | | | |
| Control | 91.84 ± 0.99 | 0.47 ± 0.54 | 7.94 ± 1.27 | 1.00 |
| Afatinib | 48.23 ± 9.13  *** | 1.84 ± 2.31 | 49.89 ± 9.52 *** | 6.28 |
| Crizotinib | 68.55 ± 1.66 *** | 3.67 ± 4.28 | 27.78 ±5.12 *** | 3.50 |
| Sorafenib | 53.02 ± 2.31  *** | 3.76 ± 5.81 | 43.28 ± 5.60 *** | 5.45 |
| Sunitinib | 58.18 ± 0.93 *** | 0.07 ± 0.12 | 41.75 ± 1.03 *** | 5.26 |

Supplementary Table 2. MKI treatment induced cellular apoptosis in Mel202, 92.1, C918 and OMM-1 cells.

Cells were pretreated with 5 μM of each MKI for 24 hours at 37°C. Cell death profile was measured with annexin V/PI flow cytometry assay. Experiments were repeated on three occasions (n=3 or 4 in each experiment). Data are presented as percentage of control (mean ± SD). *, p< 0.05; **, p<0.01; ***, p<0.001 vs. control by One-way ANOVA and Dunnett’s post-hoc test.


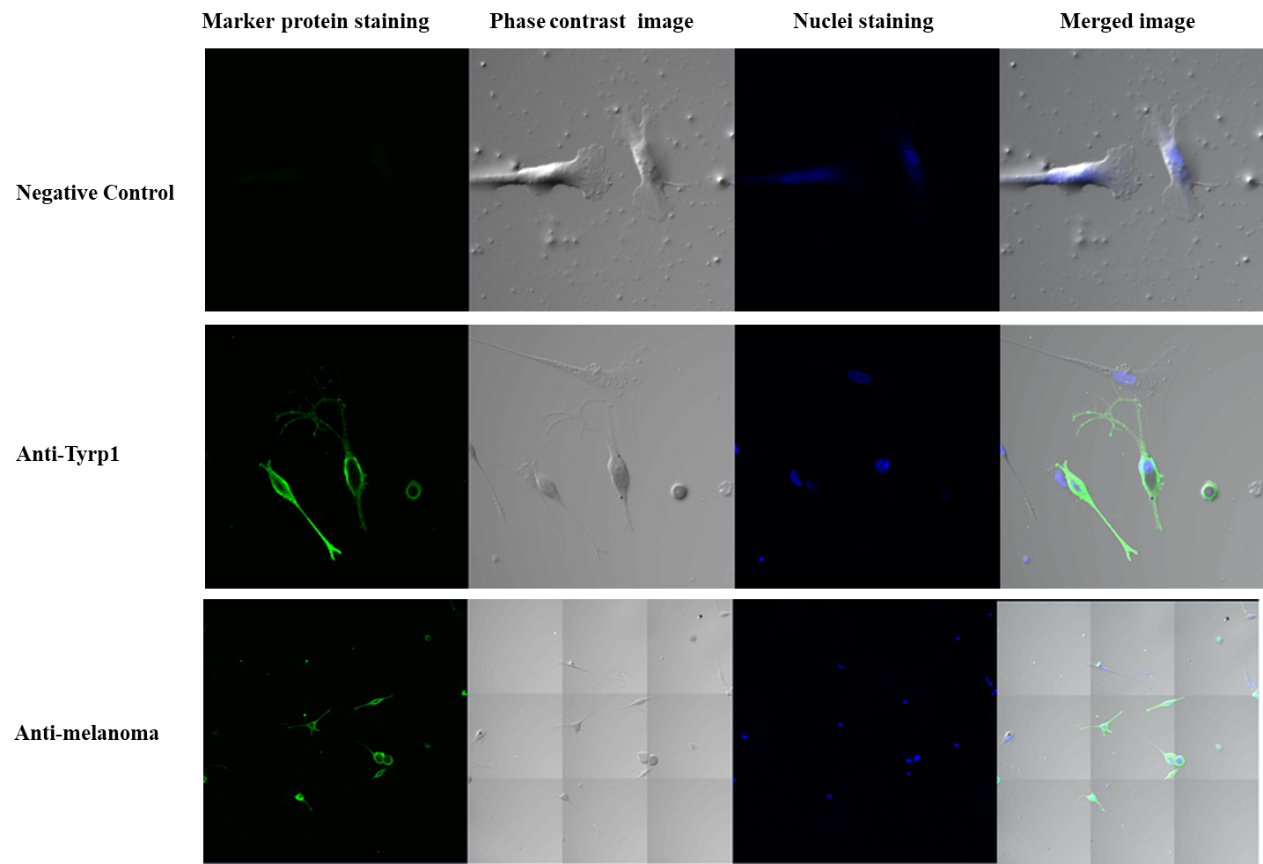


Supplementary Fig. 1 Molecular characterization of primary UM tumor-derived cell lines.

Primary UM-tumor-derived cells were cultured in chamber wells. Cells were washed, fixed and probed with or without anti-Tyrp1 (Abcam ab178676) and anti-melanoma (Abcam ab733) antibodies (1:100 dilution) overnight at 4°C. Following that the slides were washed and stained with Alexa Fluor 488 conjugated secondary antibody for 2 hr at room temperature. After washing, the slides were stained with DAPI, mounted, and sealed for imaging. The fluorescence images were taken with a confocal laser scanning microscope. Cells that were only stained with secondary antibody were adopted as the negative control. Representative images for each marker are shown in the figure. Green fluorescence indicates positive staining with anti-Tyrp1 or anti-melanoma antibodies.


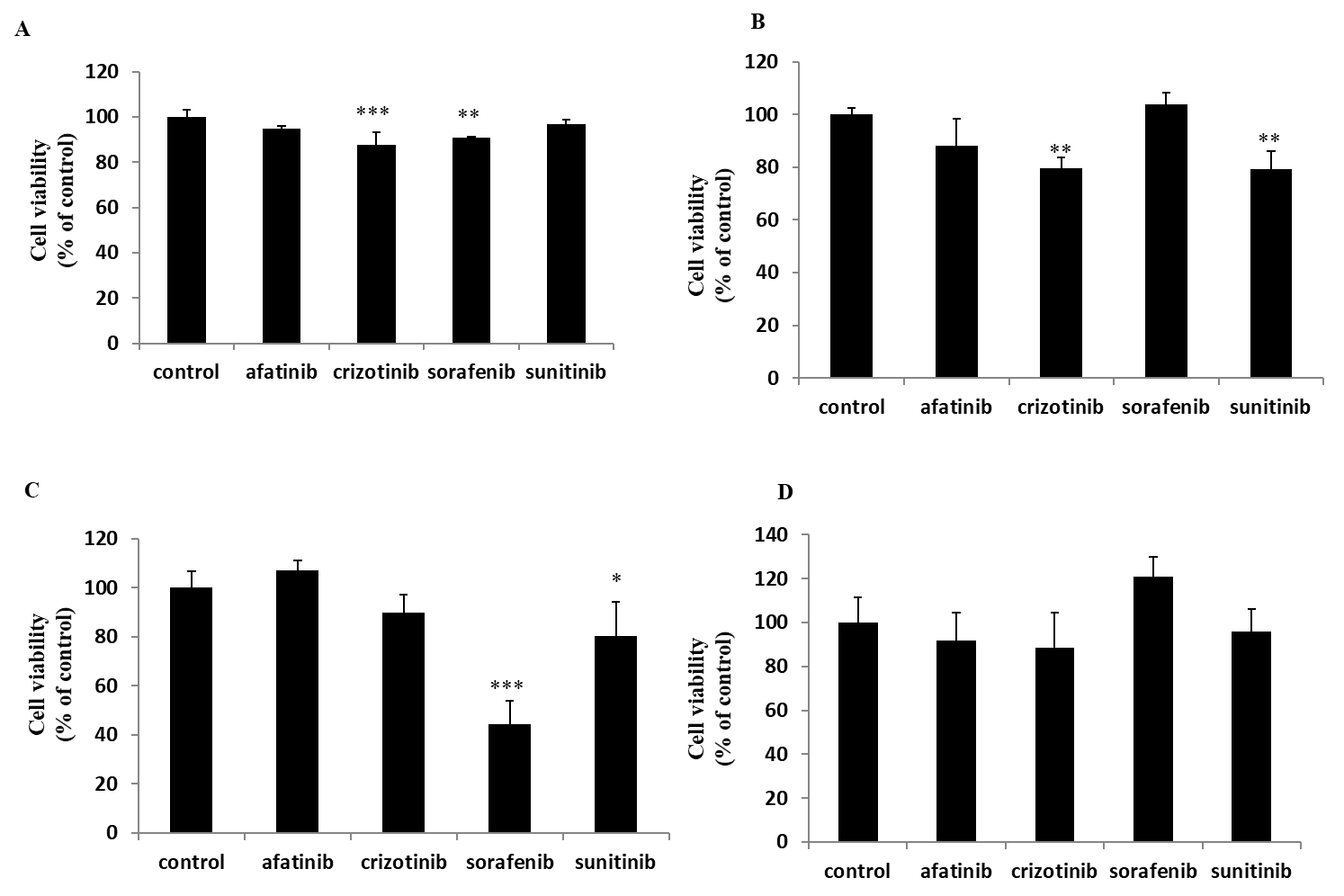


Supplementary Fig. 2 Cell viability of MIO-M1, ARPE-19 and primary cultures of human melanocyetes and fibroblasts with the treatment of MKIs

MIO-M1 (A), ARPE-19 (B) and primary cultures of human melanocyetes (C) and fibroblasts (D) were treated with each MKI (5 μM) for 24 hr at 37°C. Cell viability was assessed in cytotoxicity assays. Data are presented as percentage of control (mean ± SD). Experiments were repeated on three occasions (n=4 replicates in each experiment). *, p< 0.05; **, p<0.01; ***, p<0.001 vs. control by One-way ANOVA and Dunnett’s post-hoc test.


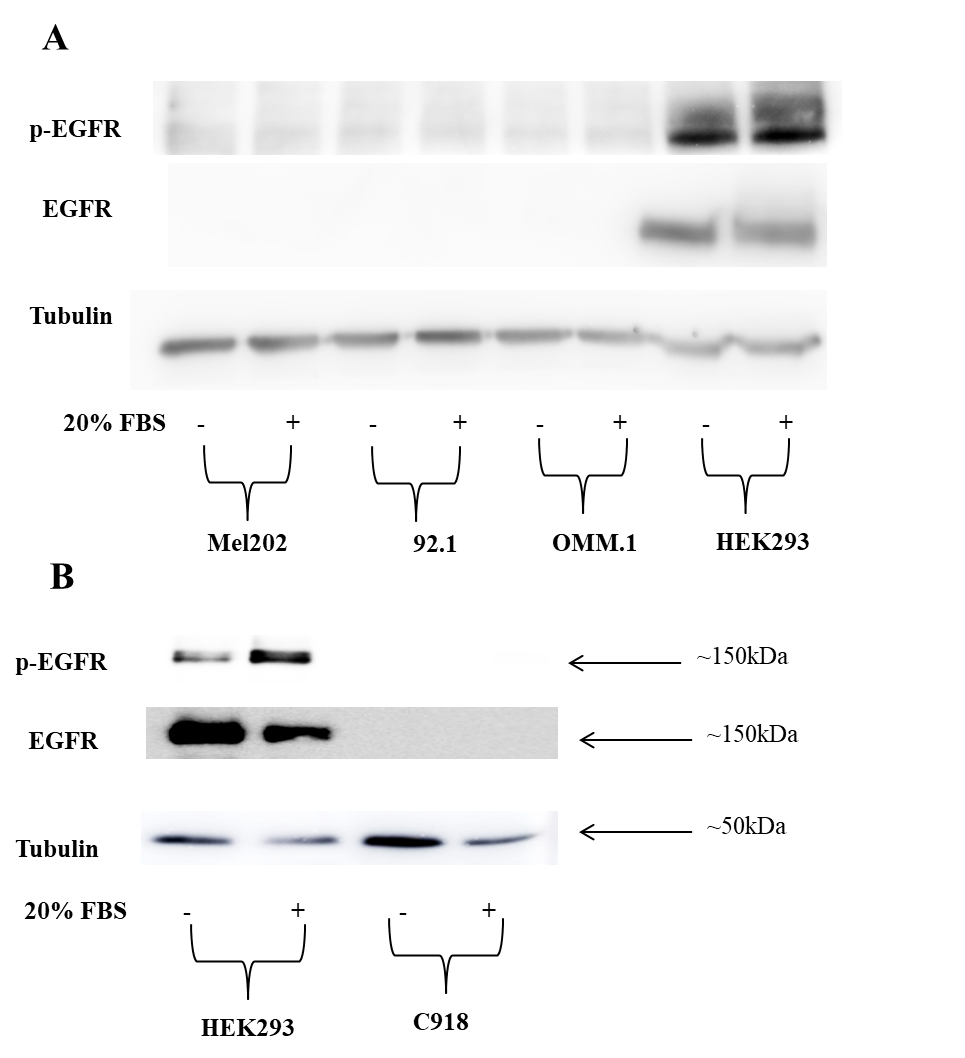


Supplementary Fig. 3 Protein expression of EGFR in human UM cell lines.

Mel202, 92.1, C918 and OMM-1 cells were starved for 24 hr at 37°C. Cells were treated acutely in the presence or absence of medium containing 20% FBS for 10 min at 37°C prior to lysis in RIPA buffer. Total cell lysates were separated on SDS-PAGE gels and protein samples were transferred to PVDF membrane. Immunoblots were probed with p-EGFR (Abcam, ab32578) and EGFR antibodies (Abcam, ab52894) at 4°C overnight (1:1000 dilution). Tubulin was used as the loading control. After washing, immunoblots were probed with HRP-conjugated secondary antibody for 2 hr at room temperature. Signals were amplified and detected with ImageQuant LAS500. Representative images are shown in the figure. The protein expression of phospho-EGFR (upper panel), EGFR (middle panel) and tubulin (bottom panel) is shown for Mel202, 92.1 and OMM-1 (A) as well as C918 (B) cells.
